# Computed tomographic features of pituitary apoplexy in a cat

**DOI:** 10.1111/jsap.13898

**Published:** 2025-06-19

**Authors:** W. Swan, M. Taylor, I. Orgonikova, J. M. De Frias

**Affiliations:** ^1^ Hospital for Small Animals, The Royal (Dick) School of Veterinary Studies The University of Edinburgh, Easter Bush Campus Midlothian UK

An 11‐year‐old, female neutered domestic long hair cat was presented, following a peracute onset of agitation, ataxia, and vocalisation progressing over three days to obtundation. Neurological examination showed severe obtundation, ambulatory tetraparesis, and compulsive circling to the left. Postural reactions were decreased on the right thoracic and pelvic limbs. There was an absent left menace response with a normal pupillary light reflex. The left eye was miotic secondary to an anterior reflex uveitis caused by a superficial corneal ulcer. The remainder of the neurological examination was unremarkable. Neuroanatomical localisation was multifocal with forebrain and brainstem lesions. Pre‐contrast computed tomography (CT) demonstrated an ovoid, slightly left lateralised hyperattenuating (~100 HU) suprasellar mass (Fig 1). Rostrally and to the right of (and confluent with) the mass was an irregularly marginated area of tissue (57 HU) that was hypoattenuating to the mass but hyperattenuating to normal brain parenchyma—this region was most consistent with recent haemorrhage. The lesions resulted in a mild mass effect on the third ventricle, hypothalamus, and midbrain, displacing them dorsally. The mass and region of suspected haemorrhage measured 7.6 mm (H) × 9 mm (W) × 5.5 mm (L). The pituitary height to brain ratio was 0.52 (reference range 0.28 ± 0.05). No CT evidence of oedema, CSF obstruction, or brain herniation was associated with the mass. Pituitary apoplexy (PA) associated with a pituitary tumour was suspected. A diffuse hepatopathy and bilateral adenomegaly were evident on abdominal ultrasound, consistent with pituitary‐dependent hyperadrenocorticism. Despite supportive treatment, the cat failed to improve, and the owner elected euthanasia. Post‐mortem examination and histopathology findings revealed a pituitary adenoma with locally extensive haemorrhage, confirming a diagnosis of PA. This case demonstrates CT imaging features of PA in a cat not previously described.

**FIG 1:**
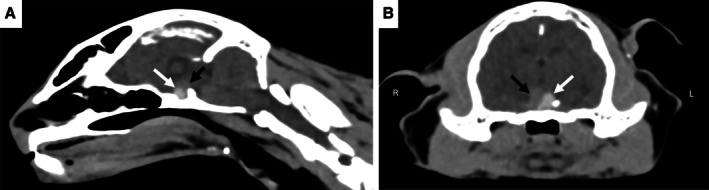
Sagittal (A) and transverse (B) CT of the head. White arrows point the suprasellar lesion, black arrows the apoplexy region.

## Author contributions

**W. Swan:** Writing – original draft; writing – review and editing. **M. Taylor:** Writing – original draft; writing – review and editing. **I. Orgonikova:** Writing – original draft; writing – review and editing. **J. M. De Frias:** Supervision; writing – review and editing; writing – original draft; conceptualization.

## Conflict of interest

No conflicts of interest have been declared.

